# Dual-Ligand Synergistic Targeting Anti-Tumor Nanoplatforms with Cascade-Responsive Drug Release

**DOI:** 10.3390/pharmaceutics15072014

**Published:** 2023-07-24

**Authors:** Fang Luo, Ting Zhong, Ying Chen, Qianqian Guo, Ling Tao, Xiangchun Shen, Yanhua Fan, Xingjie Wu

**Affiliations:** 1 State Key Laboratory of Functions and Applications of Medicinal Plants, School of Pharmaceutical Sciences, Guizhou Medical University, University Town, Guian New District, Guiyang 550025, China; 2 The Key Laboratory of Chemistry for Natural Products of Guizhou Province and Chinese Academy of Sciences, Guiyang 550014, China; 3 The High Efficacy Application of Natural Medicinal Resources Engineering Center of Guizhou Province (The Key Laboratory of Optimal Utilization of Natural Medicine Resources), School of Pharmaceutical Sciences, Guizhou Medical University, University Town, Guian New District, Guiyang 550025, China

**Keywords:** synergistic targeting, specific treatment, drug delivery, controlled release, tumor chemotherapy

## Abstract

Dual-ligand targeting drug delivery nanoplatforms are considered a promising tool for enhancing the specificity of chemotherapy. However, serious off-target delivery has been observed in current dual-ligand targeting nanoplatforms, as each ligand can independently recognize receptors on the cell membrane surface and guide drug nanocarriers to different cells. To overcome this barrier, a dual-ligand synergistic targeting (DLST) nanoplatform is developed, which can guide chemotherapy treatment specifically to cancer cells simultaneously overexpressing two receptors. This nanoplatform consists of a singlet oxygen (^1^O_2_) photosensitizer-loaded nanocarrier and a drug-loaded nanocarrier with ^1^O_2_ responsiveness, which were, respectively, decorated with a pair of complementary DNA sequences and two different ligands. For cancer cells overexpressing both receptors, two nanocarriers can be internalized in larger quantities to cause DNA hybridization-induced nanocarrier aggregation, which further activates ^1^O_2_-triggered drug release under light irradiation. For cells overexpressing a single receptor, only one type of nanocarrier can be internalized in a large quantity, leading to blocked drug release due to the ultrashort action radius of ^1^O_2_. In vivo evaluation showed this DLST nanoplatform displayed highly specific tumor treatment with minimized long-term toxicity. This is a highly efficient drug delivery system for DLST chemotherapy, holding great potential for clinical applications.

## 1. Introduction

Due to the huge normal tissue toxicity of most tumor chemotherapy drugs, highly specific drug delivery to tumor tissues has been a hot topic since the emergence of nanomedicine [1,2]. Utilizing the enhanced permeability and retention (EPR) effect of tumor tissues, drug nanocarriers ranging from 20 to 200 nm in diameter can passively accumulate in subcutaneous murine tumor tissues. However, human tumors are developed at a far slower rate, with a more complicated structure and a smaller size (relative to the host) in comparison with murine tumors, which have a largely failed EPR effect in clinical applications [3,4]. The other strategy for highly specific drug delivery is active targeting based on the interactions between the ligand at the drug nanocarrier surface and the receptors at the cancer cell membrane surface [5,6]. However, serious off-target delivery was observed in active targeting drug delivery nanoplatforms, as most tumor-related receptors can be found in various normal cells at low expression levels. To overcome this problem, three kinds of dual-ligand targeting drug delivery nanoplatforms were developed: [7,8,9,10]: (1) one that targets two different receptors on the same cancer cell surface, which can further enhance the uptake capacity of drug nanocarriers (NC); (2) one targeting cell membrane receptors and organelle receptors, which allows the NC to target specific organelles within the targeted cells after being internalized, further improving the efficacy of the drug; and (3) one targeting receptors on two different cells, such as nanodrug carriers that simultaneously target the cancer cells and endothelial cells of the tumor neovasculature. Unfortunately, directly introducing another ligand to the drug nanocarrier surface cannot relieve the off-target delivery problem, as both ligands can independently guide nanocarriers to bind with receptors on different cell membranes [11,12,13]. Therefore, a drug delivery system requiring the co-existence of two ligands in single cell to activate drug release is of great value for enhancing drug delivery specificity and pharmacological efficacy.

^1^O_2_ is a kind of highly active radical oxygen species (ROS) with a maximum action radius of 20 nm in human tissues [14,15]; it can be generated in large quantities by photosensitizers under light irradiation at a specific wavelength [16,17]. Furthermore, chemical structures with ^1^O_2_ responsiveness, including bond cleavage (such as vinyl ether, vinyl disulfide, and aminoacrylate) and hydrophobic to hydrophilic conversion (such as tellurium and imidazole), were developed for constructing drug nanocarriers with an ^1^O_2_-triggered drug release profile [18,19,20,21]. On the other hand, two nanoparticles can actively recognize each other through DNA hybridization by, respectively, anchoring a pair of complementary DNA oligos on their surfaces. Interestingly, the hybridization ability of DNA oligos anchored at the nanoparticle surface can be blocked by decorating a hydrophilic biopolymer outer layer [22,23]. In this way, controlled nanoparticle recognition can be achieved when hydrophilic biopolymers are linked to the nanoparticle surface through a stimuli-responsive bond.

**Figure 1:**
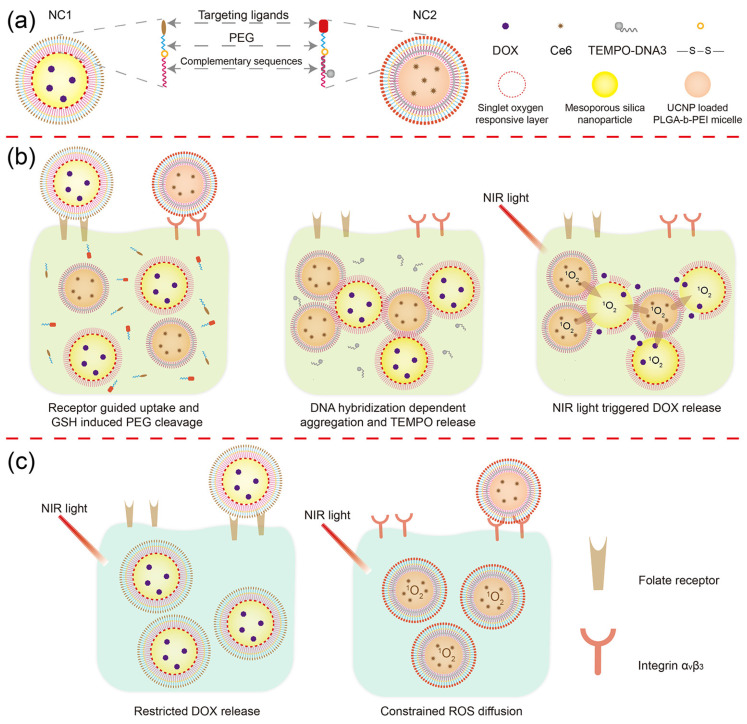
Schematic illustration of the DLST drug delivery nanoplatform. (**a**) Nanocarrier structure. The DLST nanoplatform was composed of a DOX-loaded MSN with ^1^O_2_ responsiveness (NC1) and a Ce6 + UCNP-loaded PLGA-b-PEI micelle (NC2). (**b**) DLST chemotherapy. After being internalized by a dual-receptor overexpressing tumor cell, the PEG chain will be detached from NC1 and NC2 surface in a redox tumor microenvironment. Then, NC1 and NC2 will form aggregation through DNA hybridization, accompanied by the release of TEMPO from the NC2 surface due to DNA replacement. Upon NIR light irradiation, Ce6 loaded within NC2 will generate a large quantity of ^1^O_2_ to cleave the ^1^O_2_-responsive linker and trigger the release of DOX from NC1. (**c**) Minimized normal cell toxicity. Due to the lack of intracellular GSH or the unbalanced internalization rate between NC1 and NC2, nanocarrier aggregation cannot be formed within single-receptor overexpressing cells, leading to blocked DOX release upon NIR light irradiation. Moreover, the ROS cytotoxicity was minimized by TEMPO at the NC2 surface for preserving integrin α_v_β_3_-overexpressing cells.

Inspired by the ultrashort action radius of ^1^O_2_ and steric hindrance-controlled nanoparticle recognition, we “locked” the anti-cancer drug doxorubicin (DOX) using an ^1^O_2_ responsive (SOR) linker in the first nanocarrier (NC1), and encapsulated the ^1^O_2_ photosensitizer Chlorin e6 (Ce6) with an upconverting nanoparticle (UCNP) in the second nanocarrier (NC2). After, respectively, coating a paring of total complementary DNA oligos on the NC1 and NC2 surface, polyethylene glycol-modified folic acid (PEG-FA) or PEG-modified RGD peptide (PEG-RGD) were further linked to the 5′ end of the DNA oligo through disulfide bonds (Figure 1a). For HeLa cells overexpressing both folate receptors and integrin α_v_β_3_, NC1 and NC2 can be simultaneously accumulated in HeLa tumor tissues, and can be internalized by HeLa cells in large quantities under the guidance of folic acid and RGD peptides [24,25,26]. Then, DNA hybridization-induced aggregation between NC1 and NC2 is activated by the elevated glutathione (GSH) level in the HeLa cell cytoplasm. Due to the cleavage of the SOR linker through ^1^O_2_ produced by near-infrared (NIR) light irradiation, DOX could be rapidly released from NC1 + NC2 aggregation to inhibit HeLa cell proliferation (Figure 1b). For cells overexpressing a single folate receptor, NIR light-triggered DOX release was blocked due to the ultrashort action radius of ^1^O_2_ (Figure 1c left). Meanwhile, ROS cytotoxicity for cells overexpressing the single integrin α_v_β_3_ was minimized by the ROS quencher loaded at the NC2 surface (Figure 1c right) [27]. This DLST nanoplatform requires a cascade of endogenous and exogenous stimuli to trigger drug release within cells with a specific membrane protein expression pattern, and can dramatically enhance chemotherapy specificity and pharmacological efficacy during treatment.

## 2. Materials and Methods

### 2.1. Materials

Amine-capped mesoporous silicon nanoparticles (MSN-NH_2_, diameter 100 nm) and NaYREF_4_-type UCNP were purchased from Nanjing XFNANO Materials Tech Co., Ltd. (Nanjing, China). The SOR linker, 3,3’-(ethene-1,2-diylbis(sulfanediyl)) dipropanoic acid [28], was synthesized by Shanghai NaFu Biotechnology Co., Ltd. (Shanghai, China) Singlet oxygen sensor green (SOSG) was purchased from Sigma-Aldrich. DNA oligos were synthesized by Sangon Biotech Co., Ltd. (Shanghai, China). Other reagents and materials were purchased from Shanghai Aladdin Co., Ltd. (Shanghai, China). All reagents and materials were used under the manufacture’s guidance without further purification.

### 2.2. Nanocarriers Preparation

Briefly, 1 mg MSN-NH_2_ powder and 0.5 mg DOX·HCl were incubated in 1 mL H_2_O under vigorous stirring at r. t. in the dark for 24 h to obtain DOX-loaded MSN, and were purified via centrifugation and redispersion in PBS. After each centrifugation step, the absorbance at 480 nm of the supernatant was recorded to calculate the loaded DOX amount by subtracting the unloaded DOX amount from the total DOX amount in the initial solution. Then, the excess SOR linker was reacted with 1 mg DOX-loaded MSN at r. t. for 24 h through an amidation reaction to prepare the SOR layer at the MSN surface. After that, polyethylenimine (PEI, Mw = 1800), DNA1 (AATAATAATAATAATGCCGCCGCCGCCGCC), PEG, and folic acid were sequentially coated at the SOR layer surface through amidation or Michael addition reactions to finally yield anti-cancer drug-loaded NC1. The details of the SOR layer modification process are presented in Figure S1a.

To prepare UCNP and Ce6-loaded micelle, 1 mg UCNP was mixed with 8 mg poly(lactic-co-glycolic acid) (PLGA-b-PEI, Mw = 7000) and 1 mg Ce6 in 1 mL N,N-dimethylformamide (DMF), followed by gradual addition of 2 mL water at a speed of 20 μL/min. The mixture was further incubated at r. t. under vigorous stirring overnight, and was dialyzed against H_2_O for 48 h to remove DMF and free Ce6. The amounts of loaded Ce6 and UCNP were, respectively, calculated by analyzing the Ce6 fluorescence of lyophilized micelle powder and the thermogravimetric curve of the micelle. In the next step, the micelle surface was sequentially modified using DNA2 (GGCGGCGGCGGCGGCATTATTATTATTATT), PEG, and RGD, followed by incubation with 2,2,6,6-tetramethylpiperidinyl-1-oxyl (TEMPO)-modified DNA3 to finally give NC2. The synthesis of TEMPO-DNA3 and surface modification of NC2 are illustrated in Figure S2.

### 2.3. Nanocarrier Aggregation Behavior Analysis

For the evaluation of size enlargement after aggregation, equal volumes of NC1 (1 mg/mL) and NC2 (1 mg/mL) solutions were incubated together with/without 10 mM GSH for 4 h. The obtained aggregation was centrifuged at 4000× *g* for 2 min, followed by redispersion and ultrasonication ofthe sediment in PBS for 30 min. The size of the aggregation was determined by dynamic light scattering (DLS) analysis. For analyzing aggregation kinetics, 100 μL NC1 solution (1 mg/mL), 100 μL NC2 solution (1 mg/mL), and 30 μL SYBR Green I working solution were incubated together with/without 10 mM GSH at 37 °C for different time intervals. The fluorescence intensity of SYBR Green 1 was recorded to calculate the aggregation kinetics of NC1 and NC2. To optimize the NC1: NC2 ratio for aggregation, DNA1 and DNA2 were replaced by their FAM and BHQ1-modified counterparts, respectively. Then, equal volumes of NC1 solution (1 mg/mL) and NC2 solution (gradient concentrations) were incubated with 10 mM GSH at 37 °C for 4 h. The fluorescence intensity of FAM was recorded to calculate the aggregation degree under different NC1:NC2 mass ratios.

### 2.4. In Vitro ^1^O_2_ Production and ROS Diffusion Constraints

1,3-diphenylisobenzofuran (DPBF) was used to quantify the ^1^O_2_ production of non-TEMPO-loaded NC2 under different treatments. In a typical procedure, 5 μL DPBF (1 mg/mL) was mixed with 1 mL non-TEMPO-loaded NC2 solution (1 mg/mL). Then, 50 μL of the mixture was irradiated with a 980 nm laser at a power density of 1 W/cm^2^, and the absorbance of DPBF at 410 nm was recorded every 5 min to quantify the produced ^1^O_2_ with a plate reader. The constrained ROS diffusion within NC2 was monitored via the de-esterification product of 2,7-dichlorodihydrofluorescein diacetate (DCFH-DA) [29]. Briefly, 0.5 mL DCFH-DA (1 mM, in methanol) was reacted with 2 mL NaOH (10 mM) under stirring at r. t. for 1 h. The mixture solution was neutralized to pH 7.4 by NaH_2_PO_4_ and kept at 0 °C in the dark before use. To prepare the testing medium, equal volumes of NC1 (1 mg/mL) and NC2 (1 mg/mL) were mixed together with 10 mM GSH for 4 h. Then, the freshly prepared DCFH solution was diluted with the testing medium to a final concentration of 10 μM DCFH, and was irradiated with 980 nm light (1 W/cm^2^) for different time intervals. The fluorescence intensity of DCF in the mixture solution was monitored using a plate reader.

### 2.5. NIR Light Triggered DOX Release Profile In Vitro

For monitoring the drug release profile of the NC1 + NC2 mixture, equal volumes of NC1 solution (1 mg/mL) and NC2 solution (1 mg/mL) were mixed together with/without 10 mM GSH for 4 h at 37 °C. Then, 1 mL of the mixture solution was loaded into a dialysis bag (MWCO 3500), which was further put into 5 mL PBS buffer or PBS buffer containing 10 mM GSH, and incubated at 37 °C under stirring. At predetermined time points, the PBS buffer was collected and changed periodically, and the DOX and Ce6 fluorescence of the collected PBS buffer were recorded to analyze the amount of released drugs. For analyzing the NIR light-triggered drug release profile, the mixture solution was irradiated with a 980 nm laser (power density 1 W/cm^2^) for 10 min at 2 h and 4 h. The same method was applied to monitor the independent drug release of NC1 or NC2.

### 2.6. Cell Culture

Human umbilical vein endothelial cells (HUVECs), A549 cells, and HeLa cells were obtained from the National Collection of Authenticated Cell Cultures at Shanghai, China. All cells were cultured in Dulbecco’s modified Eagle’s medium (Gibco, Grand Island, NY, USA), containing 10% FBS (Bioind, Cromwell, CT, USA) and 1% penicillin-streptomycin (Gibco).

### 2.7. Cell Internalization

The receptor-guided cell internalization of NC1 and NC2 was analyzed with a confocal laser scanning microscope (CLSM) and flow cytometry. For CLSM analyses, HeLa cells or A549 cells were cultured on coverslips in a 6-well plate at a density of 5.0 × 10^5^ cells per well for 12 h. Then, equal volumes of NC1 + NC2 mixture solution were added to HeLa cell- or A549 cell-seeded 6-well plates, which were incubated for 4 h at 37 °C. After incubation, the cells were rinsed with PBS buffer and sequentially treated with 4% polyformaldehyde for 30 min and DAPI working solution for 10 min in the dark. Finally, the coverslips were mounted and observed with a CLSM instrument. For flow cytometry analysis, the NC1 + NC2 mixture was incubated with HeLa cells or A549 cells for predetermined time intervals. After incubation, the cells were rinsed with PBS buffer, and treated with trypsin. The collected cells were analyzed using a flow cytometry instrument.

### 2.8. Intracellular ^1^O_2_ Production and ROS Constraints

SOSG and DCFH-DA were used to analyze the intracellular levels of ^1^O_2_ and ROS, respectively. In a typical procedure for ^1^O_2_ production analysis, HeLa cells cultured in a 12-well plate were incubated with a non-TEMPO-loaded NC1 + NC2 mixture (of equal NC1 and NC2 concentrations) for 6 h at 37 °C, followed by rinsing in PBS buffer three times. Then, HeLa cells were stained with 5 μL SOSG solution (10 mM) in the dark for 15 min before being irradiated with 980 nm light at a power density of 1 W/cm^2^ for 10 min. After irradiation, HeLa cells were further stained with DAPI for 10 min, and analyzed using fluorescence microscopy. For investigation of the intracellular level of ROS, HeLa cells cultured in a 12-well plate were incubated with a NC1 + NC2 mixture or their non-TEMPO-loaded counterparts for 6 h at 37 °C. Then, HeLa cells were washed with PBS and incubated with DCFH-DA for 30 min. After being irradiated with 980 nm light (1 W/cm^2^) for 10 min, the HeLa cells were stained with DAPI and analyzed using fluorescence microscopy.

### 2.9. Dual-Ligand Targeted Intracellular DOX Release

To analyze the intracellular DOX release behavior of the NC1 + NC2 mixture, HeLa cells or A549 cells cultured in 12-well plates were incubated with the NC1 + NC2 mixture (of equal NC1 and NC2 concentrations) for 6 h, followed by rinsing in PBS buffer three times. Then, the treated cells were irradiated with 980 nm light (1 W/cm^2^) for 10 min and were further incubated overnight. After incubation, the treated cells were stained with DAPI and analyzed using fluorescence microscopy. To investigate the influence of intracellular GSH concentration, the GSH synthesis of HeLa cells was blocked by incubating with 0.1 mM BSO overnight before adding the NC1 + NC2 mixture.

### 2.10. Dual-Ligand Targeted Cytotoxicity

In a typical procedure for evaluating the NIR light-triggered cytotoxicity of NC1 + NC2 mixture, HeLa cells or A549 cells cultured in a 96-well plates were treated with NC1 with gradient concentrations and NC2 with a fixed concentration (300 μg/mL) for 6 h at 37 °C, followed by 980 nm light (1 W/cm^2^) irradiation for 10 min. After being incubated for another 42 h, the viabilities of treated cells were investigated via an MTT assay. To evaluate the influence of GSH, HeLa cells were treated with 0.1 mM BSO overnight before adding the NC1 + NC2 mixture [30]. To analyze the cytotoxicity of single nanocarrier, NC1 or NC2 with gradient concentrations were incubated with HeLa cells seeded in 96-well plates for 48 h with/without 980 nm light irradiation for 10 min. To evaluate the toxicity of blank nanocarriers and NIR light irradiation, gradient concentrations of non-DOX-loaded NC1 or NC2 were incubated with HUVECs or HeLa cells for 48 h with or without 980 nm light irradiation (1 W/cm^2^) for 10 min. After incubation, the cell viabilities were investigated via an MTT assay.

### 2.11. Animal

Female Sprague Dawley rats (200–230 g) and female BALB/c nude mice (6–8 weeks, 18–20 g) were purchased from Beijing Huafukang Biotechnology Co., Ltd. (Beijing, China). All animal experiments were performed in compliance with the Guide for the Care and Use of Laboratory Animals and approved by the Animal Care Welfare Committee of Guizhou Medical University (NO. 2000674).

### 2.12. In Vivo Pharmacokinetic and Biodistribution

To investigate the pharmacokinetics of NC1 and NC2, female Sprague Dawley rats were randomly divided into four groups (*n* = 6 per group) and were administered with DOX (2 mg/kg), NC1 (equivalent to 2 mg/kg DOX), Ce6 (2 mg/kg), and NC2 (equivalent to 2 mg/kg Ce6) via the tail vein. At predetermined time points, blood samples were collected via the tail vein and were centrifuged to obtain plasma. The DOX and Ce6 in plasma were extracted and monitored by measuring their characteristic fluorescence emission at 590 nm (DOX) and 670 nm (Ce6) according to studies [31,32]. For exploring the targeted biodistribution of NC1 and NC2, HeLa, and A549, tumor-bearing female BALB/c mice were established by injecting HeLa cells or A549 cells (5 × 10^6^ cells in 200 μL PBS buffer for each mice) subcutaneously into the right flank region. When the tumor volume reached around 150 mm^3^, the mice were intravenously administered with NC1, NC2, and their none-ligand modified counterparts at equivalent DOX (2 mg/kg) and Ce6 (2 mg/kg) concentrations (6 mice per group). The in vivo fluorescence imaging of mice was carried out with a small-animal imaging system at predetermined time points. At 24 h post-injection, three mice were sacrificed to collect the major organs (heart, liver, spleen, lung, and kidney) and tumors for ex vivo fluorescence imaging.

### 2.13. In Vivo Anti-Cancer Efficacy

To evaluate the in vivo anti-cancer efficacy of the NC1 + NC2 mixture, HeLa tumor (150 mm^3^)-bearing female BALB/c mice were randomly divided into eight groups (*n* = 6) and were administered with PBS, DOX + Ce6, NC1, NC2, and NC1 + NC2 at equivalent DOX (2 mg/kg) and Ce6 (2 mg/kg) concentrations every three days via the tail vein on five occasions. For the NC1 + NIR, NC2 + NIR, and NC1 + NC2 +NIR groups, tumors were irradiated upon 980 nm light (1 W/cm^2^, 10 min) 24 h after each administration. The tumor volume [V (mm^3^) = 0.5 × length (mm) × width^2^ (mm^2^)] and mice body weight of each group were recorded every 3 days. All mice were euthanized 30 days after initial treatment. Major organs and tumors were dissected, washed with PBS and fixed in 4% polyformaldehyde for H&E, TUNEL, and PCNA analyses. To evaluate the dual-receptor-dependent in vivo anti-cancer performance of the NC1 + NC2 mixture, A549 tumor-bearing mice were treated with PBS, DOX + Ce6, NC1 + NC2, and NC1 + NC2 + NIR groups the same way as the HeLa tumor-bearing mice, and were sacrificed for histological analyses 18 days after initial treatment.

### 2.14. Biosafety Assessment

The same treatment as the anti-cancer experiment was applied to HeLa tumor-bearing mice. At 30 days after initial treatment, blood samples were collected for blood routine and blood biochemistry analyses.

### 2.15. Instrument

Absorbance and fluorescence intensity were recorded with a plate reader (Thermo Fisher Scientific, Waltham, MA, USA). The hydration diameter and surface zeta potential of the nanocarriers were analyzed with a NanoBrook 173Plus (Brookhaven Instrument, Holtsville, NY, USA). The nanocarrier morphology in dry state was analyzed via transmission electron microscopy (Talos L120C G2, Thermofisher) at 110 kV accelerating voltage without staining. The upconverting fluorescence spectrum of the NC2 solution was recorded on a steady-state and time-resolved spectrofluorometer (Edinburgh Instruments, Livingston, UK). Thermogravimetric analysis was conducted with a TGA 8000 instrument under nitrogen flow (10 mL/min) from r. t. to 900 °C at 20 °C/min. The NIR light irradiation was performed with a continuous wave diode laser (Changchun Laser Optoelectronics Technology Co., Ltd., Changchun, China). The fluorescence image and CLSM image were captured with a LEICA DMi8 (LEICA) and a LSM900 (Zeiss, Jena, Germany), respectively. Flow cytometry analysis was performed using a Nov°Cyte 2040R (ACEA), and data for 1.0 × 10^4^ gated events were collected for each experiment. The in vivo and ex vivo fluorescence images were captured bwithy an IVIS Lumina Series III (PerkinElmer, Waltham, MA, USA) at predetermined time points. The histological images of tumor and major organs were captured with a Pannoramic 250 (3DHISTECH, Budapest, Hungary) and a VS200 (OLYMPUS, Tokyo, Japan).

### 2.16. Statistics

Experiments were performed in triplicate, and the results were displayed as mean ± standard deviation. Significance evaluations were performed via a two-tailed Student’s test, and *p* < 0.05 was considered significant. The pharmacokinetic parameters were calculated using DAS 2.0 (Shanghai BioGuider Medicinal Technology Co., Ltd., Shanghai, China). Flow cytometry data were analyzed using FlowJo software FlowJo X 10.0.7r2.

## 3. Results and Discussion

### 3.1. Nanocarrier Preparation and Aggregation-Dependent Drug Release

The DLST delivery system was composed of two nanocarriers loading DOX and Ce6, respectively. For NC1, DOX was loaded within a mesoporous silica nanoparticle (MSN) capped by poly(ethylene imine) (PEI) through an SOR linker. Then, the NC1 surface was sequentially decorated with a DNA sequence (DNA1) for nanoparticle recognition and PEG-FA for active targeting (Figure S1). For NC2, photosensitizer Ce6 and UCNP (converting 980 nm light to 660 nm light) were co-loaded within the PLGA-b-PEI micelle. The purpose of loading UCNP was to minimize the potential phototoxicity of light irradiation, as NIR light is transparent to human tissue [33,34]. Similarly, the NC2 surface was sequentially decorated with the total complementary sequence (DNA2) of DNA1 as the inner layer and PEG-RGD as the outer layer (Figure S2). For preventing ROS toxicity, DNA2’s partial complementary sequence (DNA3)-modified ROS scavenger was also decorated at the NC2 surface through DNA hybridization.

**Figure 2:**
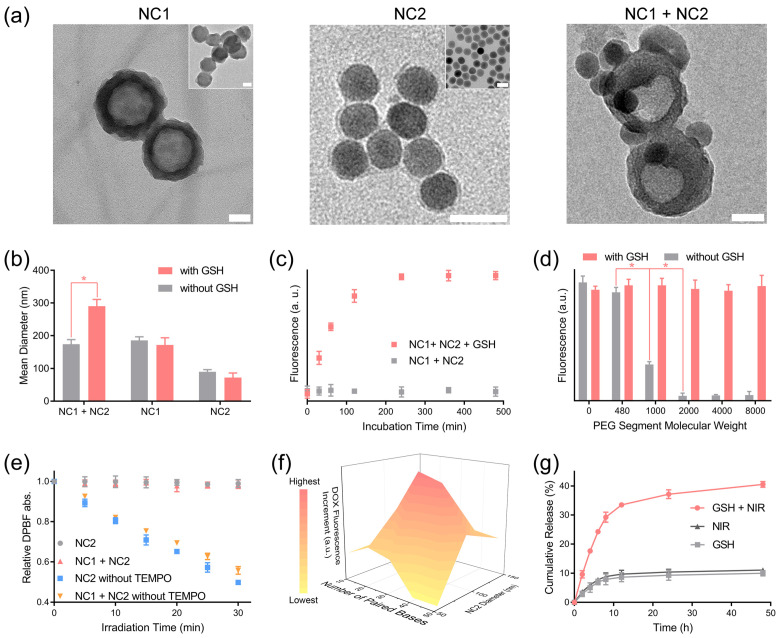
GSH-activated nanocarrier aggregation and NIR light-triggered drug release. (**a**) The morphology of NC1 (left), NC2 (middle) and their aggregation (right) were analyzed with a TEM instrument. The inset images are the unmodified MSN-NH_2_ (left, inset) and UCNP (middle inset), respectively. To induce aggregation, the NC1 + NC2 mixture was treated with GSH (10 mM) for 4 h before being deposited onto the TEM grid. Scale bar = 50 nm. (**b**) The hydration size of NC1, NC2, and the NC1 + NC2 mixture with/without 10 mM GSH treatment for 4 h. (**c**) The SYBR green fluorescence of the NC1 + NC2 mixture with/without 10 mM GSH treatment was monitored to analyze the NC1 + NC2 mixture’s aggregation kinetics. (**d**) The SYBR Green I fluorescence of NC1 + NC2 mixture modified by PEG segments with varied molecular weight with/without 10 mM GSH treatment for 4 h. (**e**) The in vitro ^1^O_2_ production of NC2, NC1 + NC2, and their counterparts without TEMPO under NIR light (980 nm, 1 W/cm^2^) irradiation over time was monitored by measuring the DPBF absorbance at 410 nm. (**f**) The DOX fluorescence increments of various NC1 + NC2 mixtures with NIR light irradiation (980 nm, 1 W/cm^2^) for 20 min relative to their counterparts without irradiation. Samples were incubated for 12 h in the dark before fluorescence analyses. (**g**) The cumulative DOX release of the NC1 + NC2 mixture with 10 mM GSH and NIR light irradiation (980 nm, 1 W/cm^2^, 20 min). Data are presented as mean ± SD and were analyzed with an unpaired two-tailed Student’s *t*-test (* *p* < 0.05).

The unmodified MSN had a spherical structure with mesoporous pores. After surface modification, a low-contrast corona structure appeared at the MSN nanoparticle periphery, with a thickness of 9.3 ± 3.2 nm, which demonstrated the formation of a functional biopolymer layer at the MSN surface (Figure 2a left). UCNP displayed a high-contrast spherical structure with a diameter of 34.8 ± 3.3 nm according to its TEM image, while a low-contrast layer (thickness 3.4 ± 0.6 nm) representing the PLGA-b-PEI micelle and surface-level coated biopolymers was observed at the UCNP periphery in the TEM image of NC2 (Figure 2a middle). After each surface modification step, both NC1 and NC2 displayed gradual hydration diameter increments (Figure S3a,c), and their surface zeta potential evolved in accordance with the electronic nature of outermost biopolymer (Figure S3b,d). More importantly, NC2 appeared at the NC1 periphery in the TEM image of the NC1 + NC2 mixture treated with 10 mM GSH (Figure 2a right), which proved that NC1 and NC2 can form aggregations in a redox tumor microenvironment [35]. Consistent with the TEM result, the hydration diameter of the NC1 + NC2 mixture enlarged drastically from 173.8 ± 14.1 nm to 290.2 ± 20.6 nm under GSH treatment, while the hydration size enlargement was not observed for the NC1 + NC2 mixture without GSH treatment (Figure 2b). In contrast, the hydration size of NC1 and NC2 shrunk slightly under GSH treatment, which was due to the cleavage of the disulfide bond and the detachment of the PEG-modified ligand from the nanocarrier surface. This further proved that the GSH-activated aggregation effect required the removal of steric hindrance at the nanocarrier surface. Furthermore, the aggregation effect can be blocked by adding free DNA1 (Figure S4a) into the NC1 + NC2 mixture solution, demonstrating that DNA hybridization was the driving force of aggregation.

SYBR Green I, capable of quantifying double-stranded DNA, was added to the NC1 + NC2 mixture to analyze the aggregation kinetics [36]. The SYBR Green I fluorescence rose gradually to a plateau in 4 h for the NC1 + NC2 mixture with GSH treatment, while the SYBR Green I fluorescence stayed constant for the non-GSH-treated NC1 + NC2 mixture (Figure 2c). Furthermore, abruptly elevated SYBR Green I fluorescence intensity was observed for the non-GSH-treated NC1 + NC2 mixtures when the PEG segment molecular weight dropped from 2000 to 1000 (Figure 2d). This indicated that a minimum PEG chain length was required to prevent inadvertent aggregation between NC1 and NC2. We also investigated the influence of the NC1 and NC2 ratio on nanocarrier aggregation, which demonstrated NC1:NC2 = 1.0:0.8 was the ideal mass ratio for a maximized aggregation effect (Figure S4b). Notably, NC1 and NC2 were stable in DMEM containing 10% fetal bovine serum (FBS) within the first 24 h incubation (Figure S4c). Additionally, comparable SYBR Green I fluorescence intensity under GSH treatment was observed for the NC1 + NC2 mixture after incubation in PBS (10% FBS) for 24 h (Figure S4d), proving that GSH-induced aggregation can be readily performed in a physiological environment.

The successful loading of DOX and Ce6 in NC1 and NC2 was confirmed by the appearance of their characteristic absorbance peaks, respectively (Figure S5a). The loading capacities of DOX (8.73%) and Ce6 (5.87%) were calculated by measuring DOX absorbance at 480 nm and Ce6 fluorescence emission at 670 nm. NC2 containing 12.6% UCNP in mass ratio with a strong upconverting fluorescence emission peak at 660 nm was applied as the ^1^O_2_-producing unit (Figure S5b,c). After being irradiated at a power density of 1 W/cm^2^ for different time intervals, the absorbance of the ^1^O_2_ indicator DPBF decreased steadily for solutions containing non-2,2,6,6-Tetramethylpiperidine 1-oxyl (TEMPO)-loaded NC2 [37], which illustrated the continuous production of ^1^O_2_ upon NIR light irradiation (Figure 2e). The produced ^1^O_2_ can be completely consumed by TEMPO loaded at the NC2 surface for the DPBF absorbance of NC2 and the NC1 + NC2 solution kept constant under NIR light irradiation for 30 min. To further confirm the constrained diffusion of other ROS produced by Ce6, the ROS concentration of the NC1 + NC2 mixture was analyzed with a fluorescence ROS probe, 2,7-dichlorofluorescein (DCFH). Negligible DCFH fluorescence enhancements were observed for the NC1 + NC2 mixture within the first 20 min of irradiation, while further elongating the irradiation time causes huge DCFH fluorescence enhancement (Figure S5d). Therefore, 20 min was set as the maximum irradiation time in this research.

Then, we investigated the influence of NC2 size and DNA hybridization length on the triggered DOX release behavior. Note NC2 with increasing hydration diameters was prepared by elongating the PLGA segment length of PLGA-b-PEI (Figure S6a). Due to the decreased curvature, NC2 with a larger hydration size experienced higher steric hindrance in the DNA hybridization at its interface. As a result, median-sized NC2 displayed significantly enhanced DOX fluorescence increments compared with the largest NC2. Interestingly, lowered DOX fluorescence increments were also observed for the smallest NC2 (diameter 53.5 nm), and were probably caused by the lowered Ce6 loading efficiency due to insufficient hydrophobic PLGA segments (Figure 2f). The ideal DNA hybridization length was found to be 30 paired bases, as a shorter or longer DNA hybridization length would destabilize aggregation or bring extra energy barriers to DNA hybridization, respectively (Table S1). This was also confirmed by the significantly enhanced SYBR Green I fluorescence for the NC1 + NC2 mixture with 30 paired bases (Figure S6b). Based on the above findings, the cumulative DOX release amount of NC1+ NC2 mixture in 48 h can be intensified by almost three-fold, to 40.5%, under the combined treatment of GSH and NIR light, in comparison with its counterparts treated with GSH (9.98%) or NIR light (11.1%) alone (Figure 2g). Despite the accelerated DOX release rate, only 4.1% Ce6 was released from the NC1 + NC2 mixture under GSH and NIR light treatment in 48 h (Figure S6c). Moreover, neither DOX nor Ce6 exhibited accelerated release from single NC1 or NC2 under the treatment of GSH and NIR light, proving the minimum DOX and Ce6 leaky release in normal tissue microenvironment (Figure S6d).

### 3.2. Targeted Intracellular DOX Release and Cytotoxicity

**Figure 3:**
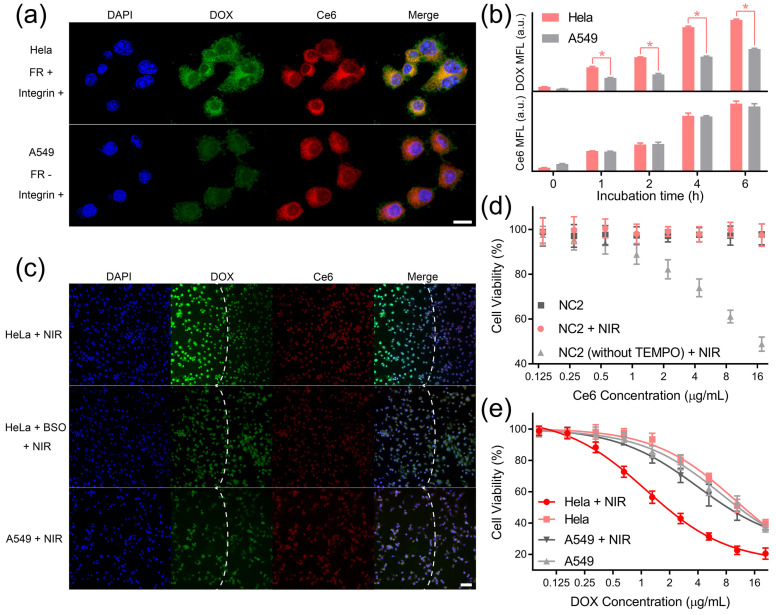
DLST intracellular DOX release and cytotoxicity. (**a**) Fluorescence images of HeLa cells (upper) and A549 cells (lower) after being incubated with the NC1 + NC2 mixture for 4 h. Scale bar = 20 μm. (**b**) Flow cytometry analyses of HeLa cells and A549 cells being treated with NC1 or NC2 at equivalent DOX and Ce6 concentrations for different time intervals. (**c**) Fluorescence images of HeLa cells, BSO-treated HeLa cells, and A549 cells 12 h after 980 nm light irradiation (1 W/cm^2^, 10 min). Laser spots are indicated by white dashed lines. Scale bar = 100 μm. (**d**) The viability of HUVECs after being incubated with NC2 and NC2 without TEMPO for 48 h with/without 980 nm irradiation (1 W/cm^2^, 10 min). (**e**) The viability of HeLa cells or A549 cells with/without 980 nm irradiation (1 W/cm^2^) for 10 min with a gradient NC1 dosage and a fixed NC2 dosage (equivalent to 17.6 mg/mL Ce6). During the treatment, cells were incubated with NC1 + NC2 mixture for 48 h in total. Data are presented as mean ± SD and were analyzed with an unpaired two-tailed Student’s *t*-test (* *p* < 0.05).

HeLa cells overexpressing both receptors and A549 cells overexpressing integrin α_v_β_3_ were, respectively, incubated with the NC1 + NC2 mixture to explore receptor-guided internalization [24,25,26]. According to the CLSM analyses, strong DOX (green) and Ce6 (red) fluorescence signals were observed upon and around the nuclei of HeLa cells, indicating the dual-receptor-accelerated internalization rate for NC1 and NC2 (Figure 3a upper). A549 cells exhibited a similar Ce6 fluorescence intensity to HeLa cells, while the DOX fluorescence of A549 cells was nearly invisible due to the absence of folate receptors at their membrane surfaces (Figure 3a, lower). Flow cytometry analyses further revealed that the DOX median fluorescence (MFL) differences between HeLa cells and A549 cells gradually enlarged over incubation time (Figure 3b upper), and the maximum MFL difference was found for cells after 4 h incubation. On the contrary, a similar Ce6 MFL was observed for HeLa cells and A549 cells (Figure 3b lower), which was in consistent with the CLSM result. Note HeLa cells and A549 cells displayed a similar cell uptake rate towards non-targeting ligand-modified NC1 and NC2, as evidenced by their fluorescence image (Figure S7a) and flow cytometry histogram (Figure S7b,c) analyses. This further confirmed that the huge difference in the NC1 internalization rate between HeLa cells and A549 cells was basically due to their different folate receptor expression levels.

Then, we tested whether the internalized NC2 can generate ^1^O_2_ to trigger DOX release in a dual-receptor-specific manner. The cellular ^1^O_2_ generation by NC2 under NIR light irradiation was confirmed by singlet oxygen sensor green (SOSG, Figure S8a), and the intracellular constraint of ROS diffusion by TEMPO was demonstrated using DCFH-DA (Figure S8b). To investigate dual-receptor-specific DOX release, the NC1 + NC2 mixture was incubated with HeLa cells or A549 cells for 6 h, and was irradiated with NIR light for 10 min. According to the fluorescence microscopy analyses, HeLa cells exhibited huge DOX signal enhancement within the light spot (Figure 3c upper), which demonstrated the triggered intracellular DOX release under NIR light irradiation. In addition, HeLa cells showed subtle DOX signal enhancement, with their intracellular GSH synthesis being inhibited by GSH (Figure 3c middle), indicating the block of DOX release in the non-tumor microenvironment. More importantly, the DOX signal was not intensified for A549 cells after NIR light irradiation because of insufficient NC1 uptake (Figure 3c lower), which clearly confirmed that the NIR light-triggered intracellular DOX release was restricted to cells overexpressing both folate receptors and integrin α_v_β_3_.

The biosafety of non-drug-loaded MSN was demonstrated by testing HUVECs’ and HeLa cells’ viability after treatment with non-drug-loaded MSN and NIR light irradiation (Figure S9a). To further confirm the eliminated ROS toxicity, NC2 was incubated with HUVECs under different treatments. Due the intracellular diffusion of highly toxic ROS, the HUVECs’ proliferation was dramatically inhibited by non-TEMPO-loaded NC2 upon NIR light irradiation. In comparison, the viability of HUVECs incubated with NC2 was well protected from ROS toxicity by TEMPO at the NC2 surface, proving the minimized normal cell toxicity of this synergistic targeting drug delivery system (Figure 3d). Notably, similar viability trends were observed for HeLa cells under the same treatment, which demonstrated that ROS toxicity can also be eliminated in the tumor microenvironment (Figure S9b). For investigating the in vitro anti-tumor performance of DOX, HeLa cells and A549 cells were treated by NC1 with gradient concentrations and NC2 with a fixed concentration (equivalent to 17.6 μg/mL Ce6). Upon NIR light irradiation, HeLa cells exhibited acute cytotoxicity with a half-maximal inhibitory concentration (IC_50_) of 2.23 μg/mL DOX, which was reduced by 81.7% relative to HeLa cells without NIR light irradiation (IC_50_ 12.21 μg/mL DOX). In comparison, NIR light irradiation could only reduce the DOX IC_50_ of A549 cells by 19.0% (from 11.16 μg/mL to 9.04 μg/mL), demonstrating significantly reduced toxicity to folate receptor-negative cells (Figure 3e). A similar phenomenon was observed for HeLa cells, with their integrin α_v_β_3_ being blocked by 10 μM RGD peptides [38,39]; their DOX IC_50_ only reduced by 13.5% (from 11.88 μg/mL to 10.28 μg/mL) under NIR light irradiation (Figure S9c). Furthermore, HeLa cells’ cyto-toxicity enhancement can also be blocked by buthionine sulfoximine (BSO) treatment (Figure S9d), which demonstrated that the redox tumor microenvironment was indispensable to activate cascade-responsive DOX release.

### 3.3. Dual-Ligand-Guided In Vivo Biodistribution

**Figure 4:**
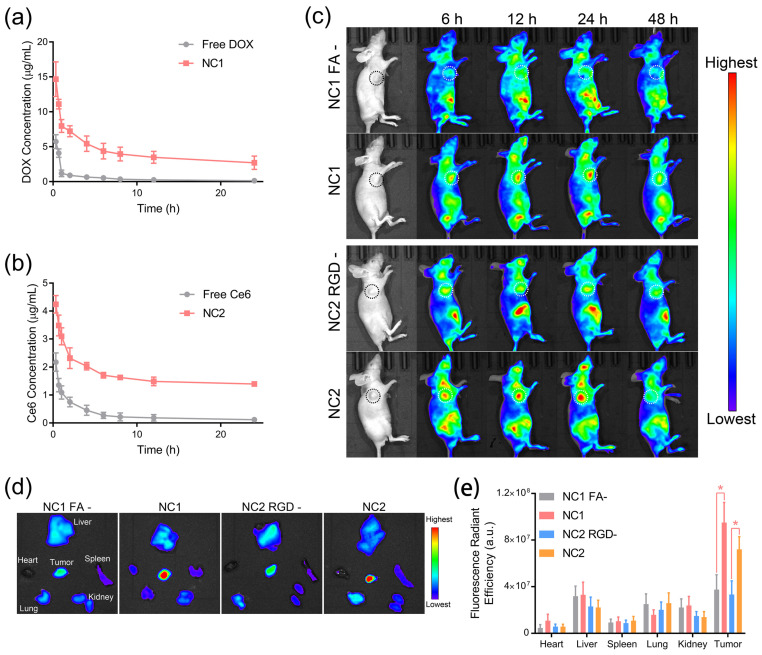
Prolonged blood circulation and ligand-guided in vivo biodistribution. (**a**,**b**) The pharmacokinetic properties of NC1 (**a**) and NC2 (**b**), as analyzed by measuring the DOX and Ce6 concentrations in plasma, respectively. (**c**) In vivo fluorescence images of HeLa tumor-bearing BALB/c nude mice treated with NC1, NC2, or their non-ligand-modified counterparts were captured to evaluate the receptor-guided biodistribution. Tumor tissues are indicated by dashed circles. (**d**) Ex vivo fluorescence images of tumor and major organs dissected from HeLa tumor-bearing nude mice. (**e**) Fluorescence radiant efficiency analyses of the DOX distribution amounts in tumors and major organs according to their ex vivo fluorescence images. Data are presented as mean ± SD and were analyzed with an unpaired two-tailed Student’s *t*-test (* *p* < 0.05).

To evaluate the pharmacokinetic properties of NC1 and NC2, female Sprague Dawley mice were intravenously administrated with nanocarriers or free drugs. As shown in Figure 4a, NC1 displayed significantly prolonged blood circulation with an elimination half-time (T_1/2_) of 2.97 h, which was 3.8 times higher than free DOX. A similar trend was observed for NC2 and free Ce6, which, respectively, possessed a T_1/2_ of 4.79 h and 1.19 h (Figure 4b). The prolonged blood circulation performances of NC1 and NC2 were also confirmed via the investigation of other pharmacokinetic parameters, including time to reach maximum concentration (T_max_), maximum concentration (C_max_), area under the curve (AUC), mean residence time (MRT) and total clearance (CL) (Table S2). Then, the tumor-targeting properties of NC1 and NC2 were analyzed using the in vivo fluorescence images of nanocarrier-treated HeLa tumor-bearing female BALB/c nude mice. Taking advantage of their prolonged nanocarrier blood circulation, NC1 and NC2-treated nude mice showed gradually enhanced fluorescence signals at the tumor site within the first 24 h after intravenous administration (Figure 4c). Notably, non-FA-modified NC1 and non-RGD-modified NC2 exhibited negligible fluorescence signals enhanced in the same time interval, indicating ligand–receptor interactions were the main driving force of nanocarrier-specific accumulation. Moreover, the fluorescence signals at the tumor site dropped dramatically for all mice 48 h after administration, which indicated the consumption of drugs by tumor tissues, as well as the clearance of circulating nanocarriers by the reticulo-endothelial system. Ex vivo fluorescence imaging experiments were performed 24 h after i. v. administration, when NC1 and NC2 possessed the maximum in vivo fluorescence signals at the tumor site. In accordance with in vivo results, HeLa tumor tissues treated with NC1 and NC2 possessed more dramatically elevated fluorescence signals than other major organs, as well as tumor tissues treated by none-ligand-modified nanocarriers (Figure 4d). On the contrary, a similar fluorescence radiant efficiency was observed in the major organs of nude mice treated with various nanocarriers (Figure 4e), which demonstrated the specificity of targeted delivery. Furthermore, increments in ex vivo fluorescence signals were only observed for A549 tumor tissues treated with NC2, while minimized fluorescence signal increments were shown for A549 tumor tissues treated with NC1 (Figure S10); these results are consistent with the A549 cell membrane protein pattern.

### 3.4. Synergistically Targeted In Vivo Tumor Treatment

**Figure 5:**
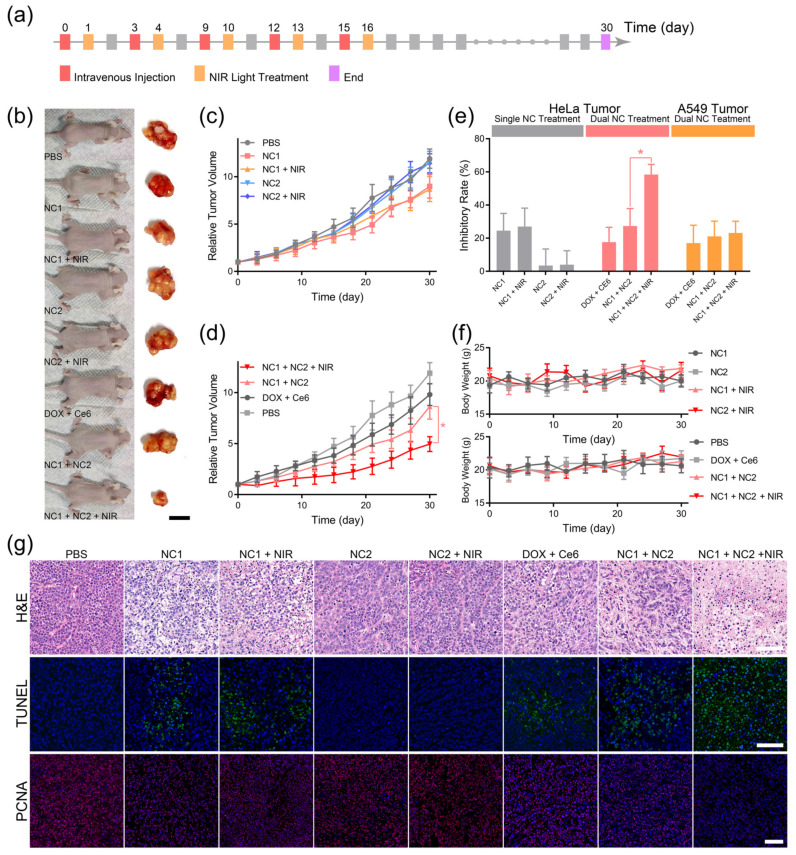
DLST anti-cancer chemotherapy. (**a**) The workflow of DLST treatment on HeLa tumor-bearing nude mice. (**b**) Representative photos of HeLa tumor-bearing nude mice and dissected tumor tissues captured at day 30. (scale bar = 1.0 cm) (**c**,**d**) Tumor volumes of single nanocarrier (**c**)- and dual nanocarrier (**d**)-treated nude mice. (**e**) The tumor inhibitory rates of HeLa tumor- and A549 tumor-bearing nude mice under various treatment. (**f**) Evolution of the nude mice’s body weights during the treatment. (**g**) H&E (upper), TUNEL (middle), and PCNA (lower) analyses of dissected HeLa tumors. (scale bar = 100 μm). Data are presented as mean ± SD (*n* = 6) and were analyzed with an unpaired two-tailed Student’s *t*-test (* *p* < 0.05).

The pharmacological efficacy of DLST tumor chemotherapy was investigated via 10 min NIR light irradiation 24 h after the i. v. injection of single nanocarrier or dual nanocarriers, and five injection and irradiation cycles were performed every 3 days to groups requiring NIR irradiation (Figure 5a). Single NC1- or NC2-treated HeLa tumor-bearing mice with/without NIR light irradiation exhibited the same approximate tumor growth rates as PBS-treated mice (Figure 5b,c), indicating minimized DOX release and constrained ROS diffusion for single-nanocarrier-accumulated tissues. In contrast, NC1 + NC2 dual-nanocarrier-treated HeLa tumor-bearing mice experienced drastically inhibited tumor growth rates under NIR light irradiation (Figure 5d), which demonstrated NIR light-triggered DOX release from NC1 + NC2 aggregation at the tumor site. Further investigation revealed that the significantly enhanced HeLa tumor inhibitory rate was induced by NIR light irradiation for NC1 + NC2-treated mice (Figure 5e). On the contrary, NIR light irradiation could not promote the A549 tumor inhibitory rate of NC1 + NC2-treated nude mice (Figure S12a–c), which clearly illustrated the high specificity of the DLST nanoplatform. In addition, neither single-nanocarrier or dual-nanocarrier-treated mice experienced body weight loss during or after the five treatment cycles, which illustrated this nanoplatform possessed minimized systemic toxicity (Figure 5f).

Some 30 days after the initial treatment cycle, all HeLa tumor-bearing mice were sacrificed for tumor and major organ histological analyses. The hematoxylin and eosin (H&E) staining of tumor tissues revealed that most tumor cells in the PBS, NC2, and NC2 + NIR groups were alive and had complete cellular structures, indicating the complete consumption of ROS by TEMPO loaded at the NC2 surface (Figure 5g upper). Furthermore, slight tumor cell shrinkage and nuclear condensation were observed for DOX + Ce6, NC1, NC1 + NIR and NC1 + NC2 groups, both of which were caused by the minimized DOX leaky release from NC1. On the contrary, significant tumor cell nuclear condensation and drastic cell density reduction were observed for the NC1 +NC2 + NIR group, showing tumor cells were undergoing necrosis due to the NIR light-triggered DOX release. TdT-mediated dUTP nick-end labeling (TUNEL) and proliferating cell nuclear antigen (PCNA) immunofluorescence staining also confirmed the NIR light-triggered DOX release from NC1 + NC2 aggregation, for only the NC1 + NC2 + NIR group exhibited a high apoptosis level with minimized proliferation of active tumor cells, while minimal tumor cell apoptosis and a large quantity of PCNA-positive tumor cells were observed for other groups (Figure 5g middle and lower). These results are inconsistent with the statistical analysis of mean fluorescence for TUNAL and PCNA images (Figure S11). However, A549 tumor-bearing mice with NC1 + NC2 + NIR treatment showed similar H&E, TUNEL, and PCNA results with other types of treatments (Figure S12d), which were in accordance with the A549 tumor growth profile and proved the efficacy of the in vivo DLST-guided treatment at cellular level.

In the final part, H&E staining of the major organs including the heart, liver, spleen, lung, and kidney was performed to evaluate the systemic toxicity of our nanoplatform. Non-pathological areas were observed for all major organs of the NC1 + NC2 and NC1 + NC2 + NIR groups (Figure S13a), suggesting ideal biosafety during the DLST treatment process. However, myocardial fiber disruption and cytoplasmic vacuolization were observed for the DOX + Ce6 group, implying cardiac injury caused by free DOX [40,41]. In addition, the blood biochemistry analyses revealed the PBS, NC1 + NC2, and NC1 + NC2 + NIR groups exhibited similar alkaline phosphatase (ALP), alanine aminotransferase (ALT), aspartate aminotransferase (AST), and blood urea levels, which demonstrated normal liver and kidney functions after treatment (Figure S13b). However, elevated ALT and AST blood concentrations were observed for the DOX + Ce6 group. As ALT and AST are both typical indicators for hepatocellular damage in clinical contexts, high ALT and AST levels implied liver damage caused by free drugs [42]. Moreover, insignificant differences were observed for routine blood indices, including hemoglobin (HGB), platelets (PLT), red blood cells (RBC), and white blood cells (WBC), for all groups, which indicated no acute toxicity of our nanoplatform (Figure S13c). Notably, the nanoplatform’s biosafety was also proven on A549 tumor-bearing mice, and the same histological results were obtained after A549 tumor treatment (Figure S14).

## 4. Conclusions

In this article, we developed DLST technology with two targeting ligands performing in a synergistic manner; we achieved this by loading a chemotherapy drug and ^1^O_2_ photosensitizer, separately, in two nanocarriers. Under NIR light irradiation, the photosensitizer-generated ^1^O_2_ may act as stimulus transducer to activate the release of drugs from the ^1^O_2_-responsive drug nanocarrier. Due to the ultra-low action radius of ^1^O_2_, the activation of drug release can only be achieved intracellularly after the formation of nanocarrier aggregation in the redox tumor microenvironment. More importantly, the ROS generated by the photosensitizer during NIR light irradiation can be consumed by TEMPO at the nanocarrier surface to prevent the ROS toxicity of single-receptor overexpressing cells. Consequently, dual-receptor-overexpressing HeLa cells exhibited a drastically reduced IC_50_ in comparison with single-receptor-overexpressing A549 cells, demonstrating DLST drug release at the cellular level. Moreover, an enhanced dual-ligand inhibitory rate against dual-receptor overexpression in HeLa tumors was achieved through NIR light irradiation, while minimal tumor inhibitory rate enhancement under NIR light irradiation was observed for single-receptor-overexpressing A549 tumors and single-nanocarrier-treated HeLa tumors. Overall, this conceptual drug delivery technology largely relieved the off-target delivery phenomenon caused by the ubiquitous expression of pathological receptors and unspecific cell internalization through the non-receptor-guided pathway. Taking advantage of its high specificity at the cellular level, this DLST technology can be applied to (1) construct delivery systems with enhanced drug pharmacological efficacy and potentially reduced multi-drug resistance, and (2) develop a treatment modality for extremely fragile and vital organs, in which cases the preservation of normal cells and tissue functionalities is of great value.

## Data Availability

The data that support the findings of this study are available within this article and its Supplementary Information, or from the corresponding author upon reasonable request.

## Supplementary Materials

The following supporting information can be downloaded at: https://www.mdpi.com/article/10.3390/pharmaceutics15072014/s1, Figure S1: Schematic illustration of NC1 surface modification. The surface of MSN-NH_2_ was sequentially modified with excess singlet oxygen-responsive (SOR) linkers and PEI (M_W_ 600). Then, DNA1 (10 μM) was modified at the MSN surface through the aza-Michael addition between the acrylate moiety at the 3′ end of DNA1 and the primary amine of PEI. Finally, excess dicystamine-modified PEG and folic acid were sequentially coated to the MSN surface through an amidation reaction to yield NC1 for the DLST anti-cancer treatment. After each step, MSN was purified via centrifugation and redispersion in PBS; Figure S2: (a) The synthesis route of TEMPO-DNA3. In the first step, DNA3 with 5′ acrylate was reacted with excess tris(2-aminoethyl)amine in PBS for 24 h. Then, the obtained DNA3 with 5′ bisamine was incubated with excess N,N′-Methylenedacrylamide for 24 h in PBS to give DNA3 with 5′ diacrylate. Finally, DNA3 (GCCGCCGCCGCCGCC) with 5′ diacrylate was further reacted with NH_2-_TEMPO to obtain TEMPO-DNA3. After each reaction, DNA3 was purified with a centrifugal filter (Amicon). (b) Schematic illustration of NC2 surface modification. After forming UCNP and Ce6-loaded micelle through a self-assembly technique in water, the primary amine of the PEI segment was reacted with an acrylate moiety at the 3′ end of DNA2 (10 μM). Then, excess cystamine and PEG diacrylate were sequentially linked to the micelle surface through amidation and aza-Michael addition, respectively. Finally, the micelle was incubated with RGD peptide and TEMPO-DNA3 to obtained NC2. After each reaction, micelles were purified with a centrifugal filter to remove residual reagents (Amicon); Figure S3: The hydration size and surface zeta potential of NC1 and NC2 during surface modification processes were recorded with a dynamic light scattering instrument. Gradually enlarged hydration diameters were observed for the MSN-NH_2_ (a) and UCNP + Ce6-loaded micelle (UCNP@Ce6@M) (b) after each surface modification step. The surface zeta potentials of MSN-NH_2_ (c) and UCNP@Ce6@M (d) changed from positive to negative after modification of the DNA1 and DNA2 at their surface, respectively; Figure S4: (a) The NC1 + NC2 mixture and DNA1 were incubated together with 10 mM GSH for 4 h. A steadily shrinking hydration size of the NC1 + NC2 mixture was observed with increasing DNA1 concentration. (b) DNA1 and DNA2 coated at NC1 and NC2 were, respectively, replaced by their counterpart sequences modified with FAM or BHQ. The FAM fluorescence of the NC1 + NC2 mixture with 1 mg/mL NC1 and gradient NC2 concentration was recorded to optimize the NC1 and NC2 ratio for aggregation under GSH treatment. (c) NC1 and NC2 were incubated in PBS buffer containing 10% FBS for 48 h at 37 ^o^C. In the first 24 h, (d) after being incubated in PBS buffer (10% FBS), NC1 + NC2 mixtures were treated with 10 mM GSH and SYBR Green I. Similar SYBR Green fluorescence was observed for the NC1 + NC2 mixtures under 10% FBS treatment within 24 h; Figure S5: (a) The characteristic peaks of DOX and Ce6 appeared on the absorbance spectrum of the NC1 and NC2 solution, respectively. (b) Strong emission intensities at 550 nm and 660 nm were observed on the upconverting fluorescence spectrum of the UCNP-loaded micelle solution and NC2 solution. (c) The lyophilized powder of NC2 and its non-UCNP-loaded counterpart were analyzed with a TGA instrument. The UCNP content was calculated to be 12.6% for NC2 according to the TGA analyses results. (d) The fluorescence intensity of DCFH (a ROS probe) in NC1 + NC2 mixture solution with different NIR light (980 nm, 1 W/cm^2^) irradiation times; Figure S6: (a) Hydration diameters of NC2 prepared from PLGA-b-PEI with elongated PEI segment length. (b) SYBR Green I fluorescence of the NC1 + NC2 mixture with different numbers of paired bases. (c) The Ce6 cumulative release profile of the NC1 + NC2 mixture under 10 mM GSH treatment, NIR light irradiation (980 nm, 1W/cm^2^) for 20 min, or the combined treatment of GSH and NIR light. (d) The 48 h cumulative release of DOX or Ce6 from single NC1 or NC2 in 10 mM GSH with/without NIR light irradiation (980 nm, 1 W/cm^2^) for 20 min; Figure S7: (a) Fluorescence image of HeLa cells (upper) and A549 cells (lower) after being incubated with the NC1 + NC2 mixture for 4 h. Scale bar = 100 μm. (b) Flow cytometry histogram of HeLa cells and A549 cells after being incubated with NC1 (PE-H) or NC2 (APC-H); Figure S8: (a) Fluorescence images of non-TEMPO-loaded NC2-treated HeLa cells under NIR light (980 nm, 1 W/cm^2^) irradiation for 0 and 10 min. HeLa cells were stained with DAPI and ^1^O_2_ probe SOSG. Scale bar = 200 μm. (b) Fluorescence images of NC2 or its non-TEMPO-loaded counterpart-treated HeLa cells under NIR light irradiation (980 nm, 1 W/cm2) for 10 min. HeLa cells were stained with DAPI and ROS probe DCFH-DA. Scale bar = 200 μm; Figure S9: (a) The viability of HUVECs and HeLa cells after being treated with non-DOX-loaded NC1 for 48 h with/without NIR light irradiation (980 nm, 1W/cm^2^) for 10 min. (b) The viability of HeLa cells under 10 min NIR light irradiation after being incubated with NC2 or its non-TEMPO-loaded counterpart. (c) The viability of free RGD (10 μm) and NC1 + NC2 mixture-treated HeLa cells with/without NIR light irradiation. (d) The viability of BSO (0.1 mM) and NC1 + NC2 mixture-treated HeLa cells with/without NIR light irradiation; Figure S10: Ex vivo fluorescence image of tumors and major organs collected from A549 tumor-bearing nude mice after being treated with NC1 and NC2 or their non-ligand-modified counterparts for 24 h; Figure S11: The mean fluorescence of TUNEL (a) and PCNA (b) images (* indicates significant difference from others, *p < 0.05*); Figure S12: (a) Representative photos of A549 tumor-bearing BALB/c nude mice 18 days after the initial treatment with PBS, DOX + Ce6, NC1 + NC2, or NC1 + NC2 + NIR at an equivalent dosage of 2 mg/kg DOX and 2 mg/kg Ce6. All nude mice were subjected to five injections. For the NC1 + NC2 + NIR group, nude mice were irradiated with NIR light (980 nm, 1 W/cm^2^, 10 min) 24 h after each injection. The body weight (b) and tumor volume (c) of A549 tumor-bearing nude mice were recorded every 3 days during the treatment. (d) A549 tumor-bearing nude mice were sacrificed 18 days after initial treatment. The dissected tumor tissues were subjected to H&E, TUNEL, and PCNA analyses. Scale bar = 100 μm. Data are presented as mean ± SD (n = 6); Figure S13: Biosafety evaluation. (a) The major organs of HeLa tumor-bearing nude mice after treatment were subjected to H&E staining. No obvious toxicities were induced by the NC1 + NC2 and NC1 + NC2 + NIR groups in all major organs. In comparison, cardiac injury was observed for DOX + Ce6-treated nude mice. (b,c) Blood samples of tumor-bearing nude mice were collected after treatment for blood biochemistry (c) and blood routine (d) analyses. Similar ALP, ALT, AST, and BUN levels were observed for PBS, NC1 + NC2, and NC1 + NC2 + NIR-treated nude mice. For DOX + Ce6-treated mice, elevated AST levels, indicating hepatic toxicity, were observed. HeLa tumor-bearing nude mice exhibited similar blood routine indices, including HGB, PLT, RBC, and WBC, for all groups. Data are presented as mean ± SD and were analyzed with an unpaired two-tailed Student’s *t*-test (* *p* < 0.05); Figure S14: H&E analyses results of major organs dissected from A549 tumor-bearing nude mice after various treatments. Scale bar = 100 μm; Table S1: DNA1 and DNA2 sequences with a different number of paired bases used to optimize NIR light-triggered DOX release; Table S2: Pharmacokinetic parameters of NC1 and NC2.
