# Supplementary material for: Dual-Ligand Synergistic Targeting Anti-Tumor Nanoplatforms with Cascade-Responsive Drug Release
Source: Pharmaceutics. 2023 Jul 24;15(7):2014. doi: 10.3390/pharmaceutics15072014 (PMC10385531; doi:10.3390/pharmaceutics15072014)
Supplement: Supplementary file 1 [file pharmaceutics-15-02014-s001.zip › pharmaceutics-2457996-supplementary.pdf]

Supplementary data for

Dual-Ligand Synergistic Targeting Anti-Tumor Nanoplatforms with Cascade-Responsive Drug Release

Fang Luo, Ting Zhong, Ying Chen, Qianqian Guo, Ling Tao, Xiangchun Shen *,

Yanhua Fan * and Xingjie Wu *

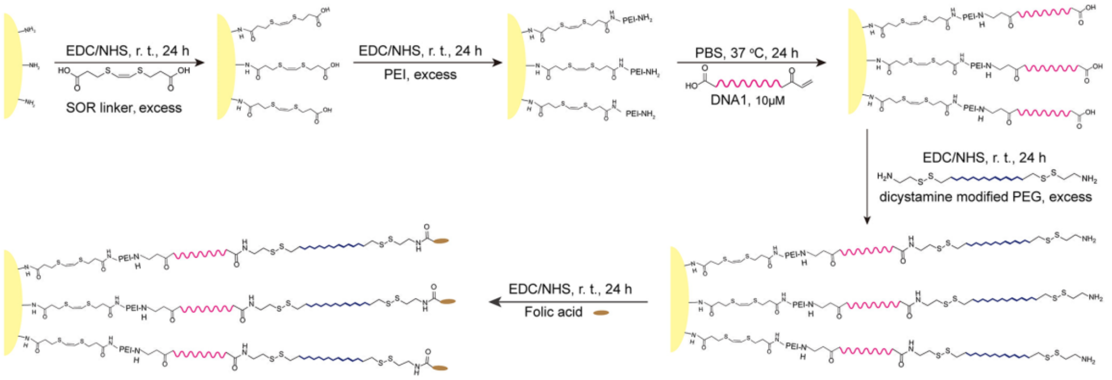

Figure S1. Schematic illustration of NC1 surface modification. The surface of MSN-NH_2 was sequentially modified with excess singlet oxygen-responsive (SOR) linkers and PEI (M_W 600). Then, DNA1 (10 μM) was modified at the MSN surface through the aza-Michael addition between the acrylate moiety at the 3` end of DNA1 and the primary amine of PEI. Finally, excess dicystamine-modified PEG and folic acid were sequentially coated to the MSN surface through an amidation reaction to yield NC1 for the DLST anti-cancer treatment. After each step, MSN was purified via centrifugation and redispersion in PBS.

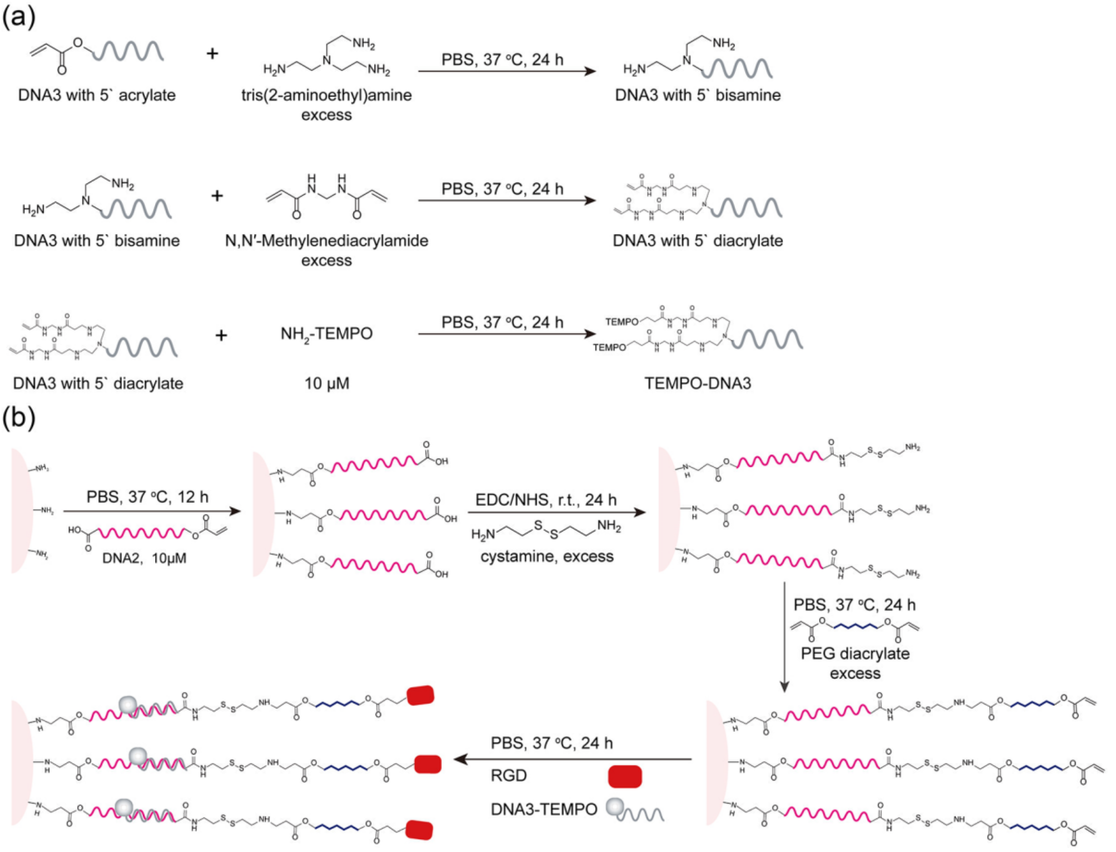

Figure S2. (**a**) The synthesis route of TEMPO-DNA3. In the first step, DNA3 with 5` acrylate was reacted with excess tris(2-aminoethyl)amine in PBS for 24 h. Then, the obtained DNA3 with 5` bisamine was incubated with excess N,N`-Methylenedacrylamide for 24 h in PBS to give DNA3 with 5` diacrylate. Finally, DNA3 (GCCGCCGCCGCCGCC) with 5` diacrylate was further reacted with NH_2-TEMPO to obtain TEMPO-DNA3. After each reaction, DNA3 was purified with a centrifugal filter (Amicon). (**b**) Schematic illustration of NC2 surface modification. After forming UCNP and Ce6-loaded micelle through a self-assembly technique in water, the primary amine of the PEI segment was reacted with an acrylate moiety at the 3` end of DNA2 (10 μM). Then, excess cystamine and PEG diacrylate were sequentially linked to the micelle surface through amidation and aza-Michael addition, respectively. Finally, the micelle was incubated with RGD peptide and TEMPO-DNA3 to obtained NC2. After each reaction, micelles were purified with a centrifugal filter to remove residual reagents (Amicon).

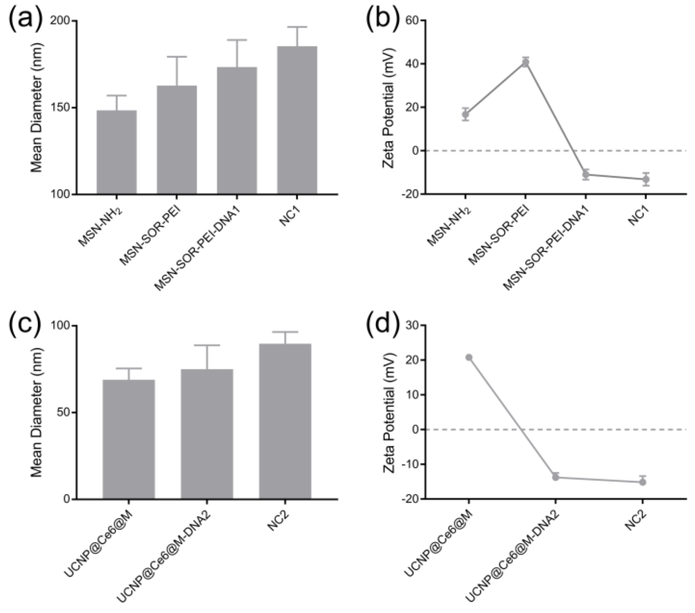

Figure S3. The hydration size and surface zeta potential of NC1 and NC2 during surface modification processes were recorded with a dynamic light scattering instrument. Gradually enlarged hydration diameters were observed for the MSN-NH_2 (**a**) and UCNP + Ce6-loaded micelle (UCNP@Ce6@M) (**b**) after each surface modification step. The surface zeta potentials of MSN-NH_2 (**c**) and UCNP@Ce6@M (**d**) changed from positive to negative after modification of the DNA1 and DNA2 at their surface, respectively.

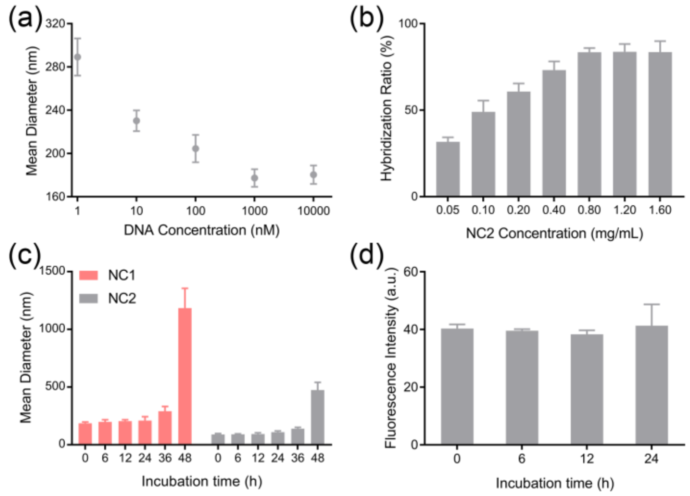

Figure S4. (**a**) **The** NC1 + NC2 mixture and DNA1 were incubated together with 10 mM GSH for 4 h. A steadily shrinking hydration size of the NC1 + NC2 mixture was observed with increasing DNA1 concentration. (**b**) DNA1 and DNA2 coated at NC1 and NC2 were, respectively, replaced by their counterpart sequences modified with FAM or BHQ. The FAM fluorescence of the NC1 + NC2 mixture with 1 mg/mL NC1 and gradient NC2 concentration was recorded to optimize the NC1 and NC2 ratio for aggregation under GSH treatment. (**c**) NC1 and NC2 were incubated in PBS buffer containing 10% FBS for 48 h at 37 °C. In the first 24 h, (**d**) after being incubated in PBS buffer (10% FBS), NC1 + NC2 mixtures were treated with 10 mM GSH and SYBR Green I. Similar SYBR Green fluorescence was observed for the NC1 + NC2 mixtures under 10% FBS treatment within 24 h.

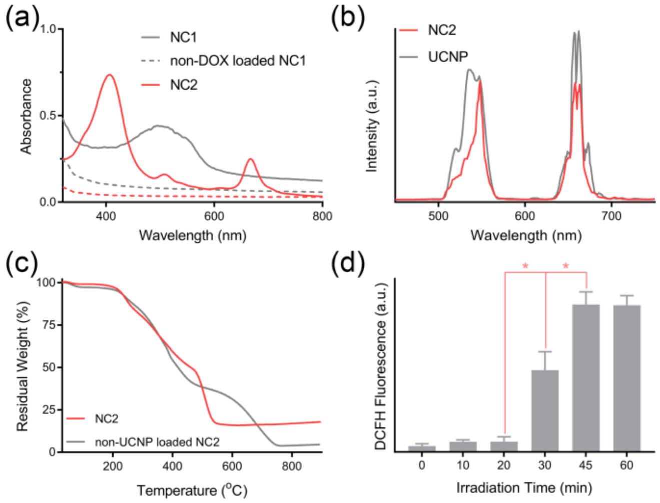

Figure S5. (**a**) The characteristic peaks of DOX and Ce6 appeared on the absorbance spectrum of the NC1 and NC2 solution, respectively. (**b**) Strong emission intensities at 550 nm and 660 nm were observed on the upconverting fluorescence spectrum of the UCNP-loaded micelle solution and NC2 solution. (**c**) The lyophilized powder of NC2 and its non-UCNP-loaded counterpart were analyzed with a TGA instrument. The UCNP content was calculated to be 12.6 % for NC2 according to the TGA analyses results. (**d**) The fluorescence intensity of DCFH (a ROS probe) in NC1 + NC2 mixture solution with different NIR light (980 nm, 1 W/cm^2) irradiation times.

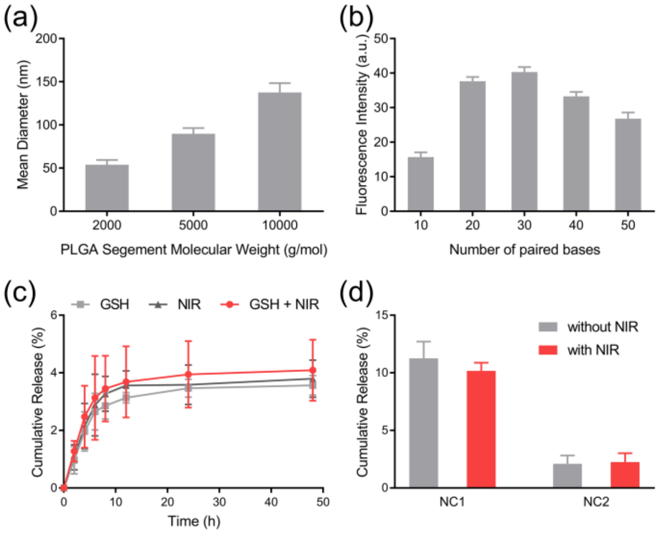

Figure S6. (**a**) Hydration diameters of NC2 prepared from PLGA-b-PEI with elongated PEI segment length. (**b**) SYBR Green I fluorescence of the NC1 + NC2 mixture with different numbers of paired bases. (**c**) The Ce6 cumulative release profile of the NC1 + NC2 mixture under 10 mM GSH treatment, NIR light irradiation (980 nm, 1W/cm^2) for 20 min, or the combined treatment of GSH and NIR light. (**d**) The 48 h cumulative release of DOX or Ce6 from single NC1 or NC2 in 10 mM GSH with/without NIR light irradiation (980 nm, 1 W/cm^2) for 20 min.

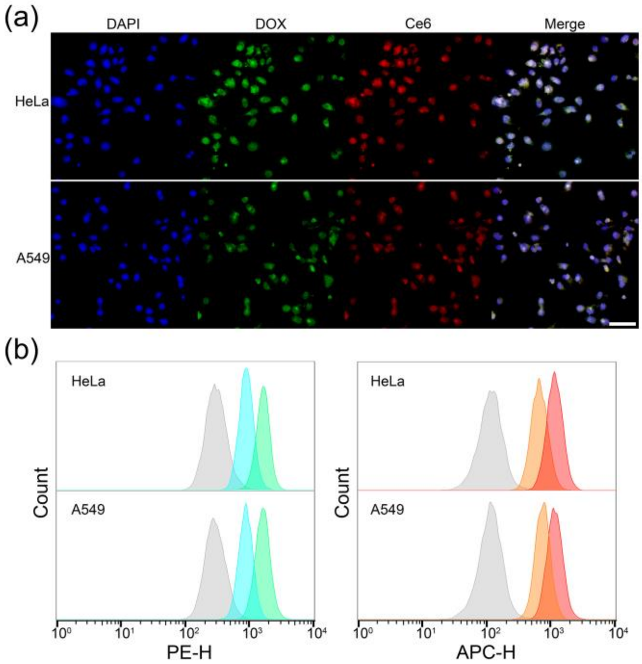

Figure S7. (**a**) Fluorescence image of HeLa cells (upper) and A549 cells (lower) after being incubated with the NC1 + NC2 mixture for 4 h. Scale bar = 100 μm. (**b**) Flow cytometry histogram of HeLa cells and A549 cells after being incubated with NC1 (PE-H) or NC2 (APC-H).

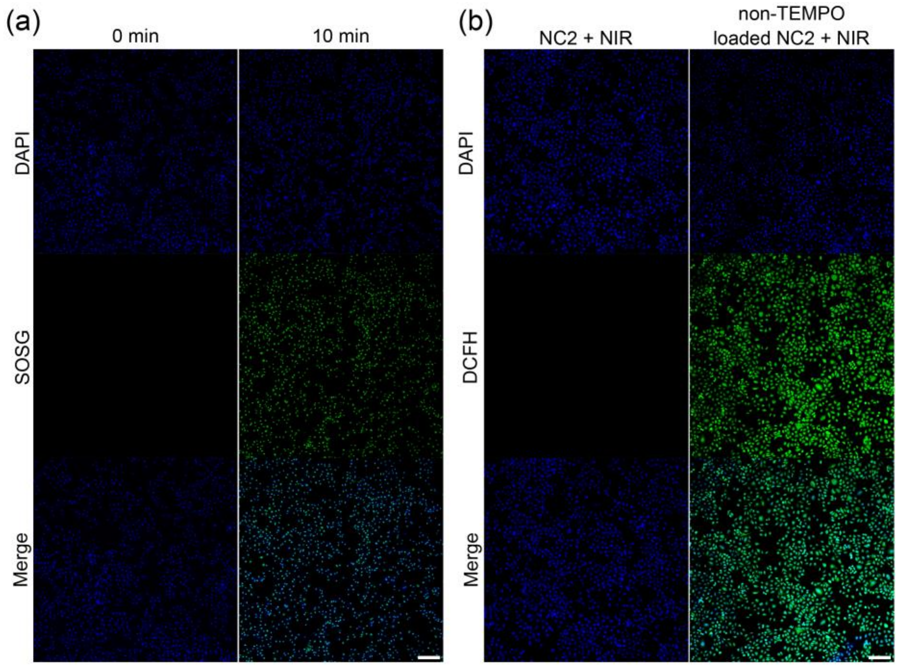

Figure S8. (**a**) Fluorescence images of non-TEMPO-loaded NC2-treated HeLa cells under NIR light (980 nm, 1 W/cm^2) irradiation for 0 and 10 min. HeLa cells were stained with DAPI and $^1O_2$ probe SOSG. Scale bar = 200 μm. (**b**) Fluorescence images of NC2 or its non-TEMPO-loaded counterpart-treated HeLa cells under NIR light irradiation (980 nm, 1 W/cm2) for 10 min. HeLa cells were stained with DAPI and ROS probe DCFH-DA. Scale bar = 200 μm.

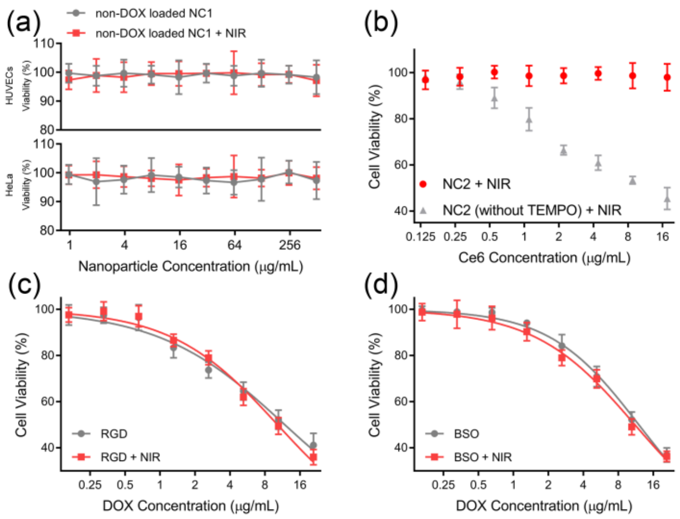

Figure S9. (**a**) The viability of HUVECs and HeLa cells after being treated with non-DOX-loaded NC1 for 48 h with/without NIR light irradiation (980 nm, 1W/cm^2) for 10 min. (**b**) The viability of HeLa cells under 10 min NIR light irradiation after being incubated with NC2 or its non-TEMPO-loaded counterpart. (**c**) The viability of free RGD (10 μm) and NC1 + NC2 mixture-treated HeLa cells with/without NIR light irradiation. (**d**) The viability of BSO (0.1 mM) and NC1 + NC2 mixture-treated HeLa cells with/without NIR light irradiation.

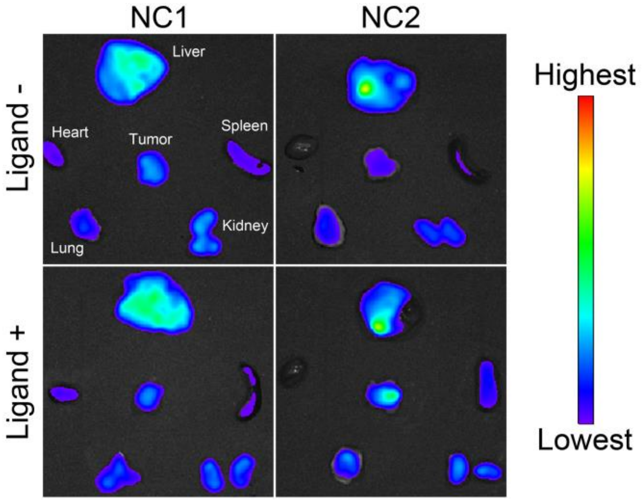

Figure S10. Ex vivo fluorescence image of tumors and major organs collected from A549 tumor-bearing nude mice after being treated with NC1 and NC2 or their non-ligand-modified counterparts for 24 h.

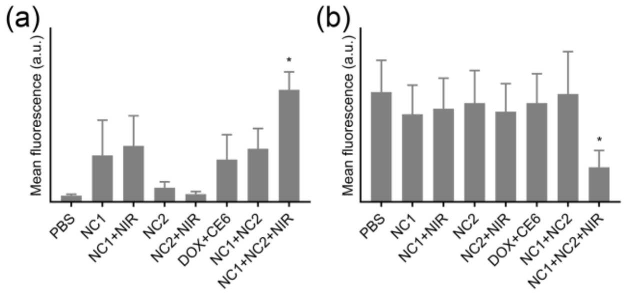

Figure S11. The mean fluorescence of TUNEL (**a**) and PCNA (**b**) images (* indicates significant difference from others, $p < 0.05$).

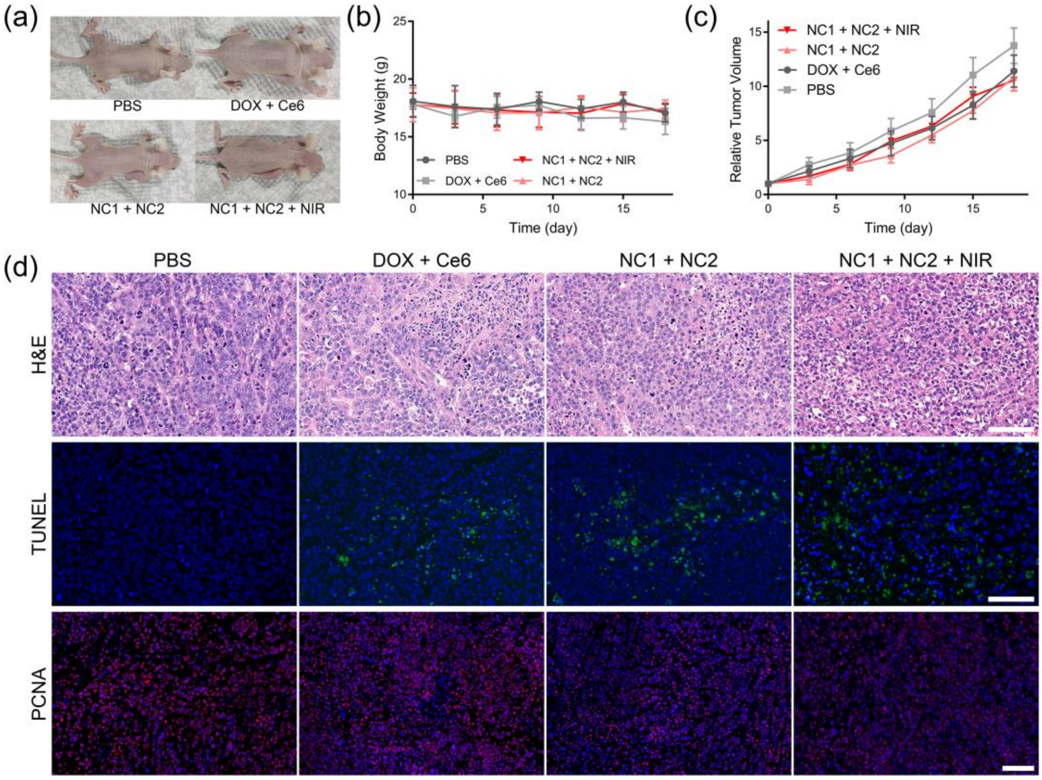

Figure S12. (**a**) Representative photos of A549 tumor-bearing BALB/c nude mice 18 days after the initial treatment with PBS, DOX + Ce6, NC1 + NC2, or NC1 + NC2 + NIR at an equivalent dosage of 2 mg/kg DOX and 2 mg/kg Ce6. All nude mice were subjected to five injections. For the NC1 + NC2 + NIR group, nude mice were irradiated with NIR light (980 nm, 1 W/cm^2, 10 min) 24 h after each injection. The body weight (**b**) and tumor volume (**c**) of A549 tumor-bearing nude mice were recorded every 3 days during the treatment. (**d**) A549 tumor-bearing nude mice were sacrificed 18 days after initial treatment. The dissected tumor tissues were subjected to H&E, TUNEL, and PCNA analyses. Scale bar = 100 μm. Data are presented as mean ± SD (n = 6).

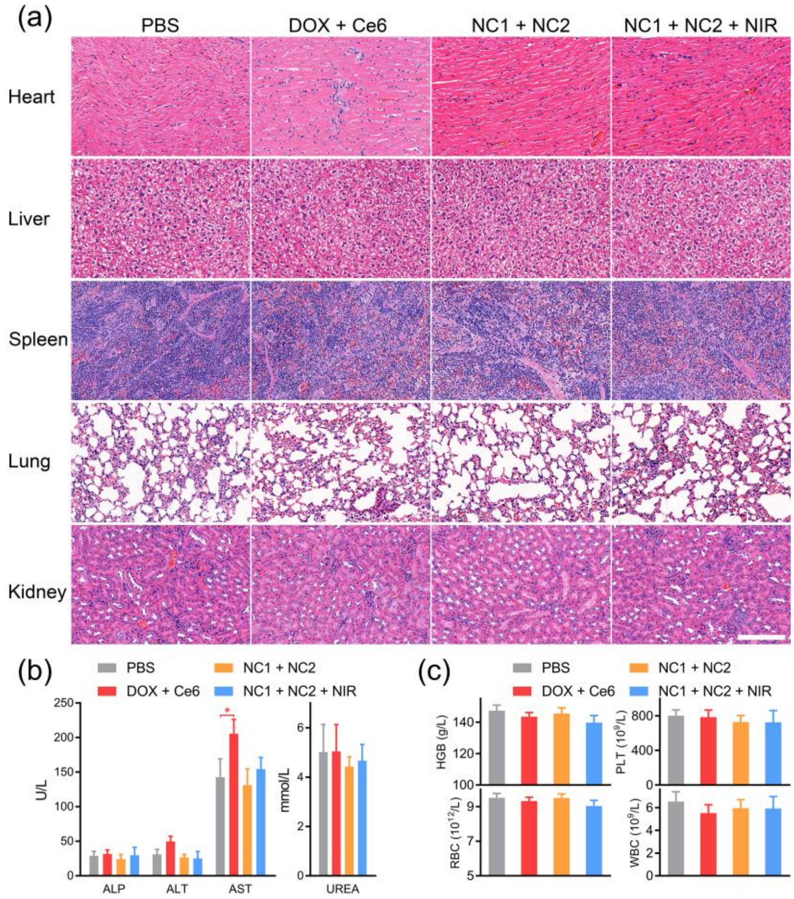

Figure S13. Biosafety evaluation. (**a**) The major organs of HeLa tumor-bearing nude mice after treatment were subjected to H&E staining. No obvious toxicities were induced by the NC1 + NC2 and NC1 + NC2 + NIR groups in all major organs. In comparison, cardiac injury was observed for DOX + Ce6-treated nude mice. (**b**,**c**) Blood samples of tumor-bearing nude mice were collected after treatment for blood biochemistry (c) and blood routine (d) analyses. Similar ALP, ALT, AST, and BUN levels were observed for PBS, NC1 + NC2, and NC1 + NC2 + NIR-treated nude mice. For DOX + Ce6-treated mice, elevated AST levels, indicating hepatic toxicity, were observed. HeLa tumor-bearing nude mice exhibited similar blood routine indices, including HGB, PLT, RBC, and WBC, for all groups. Data are presented as mean ± SD and were analyzed with an unpaired two-tailed Student's *t*-test (* $p < 0.05$).

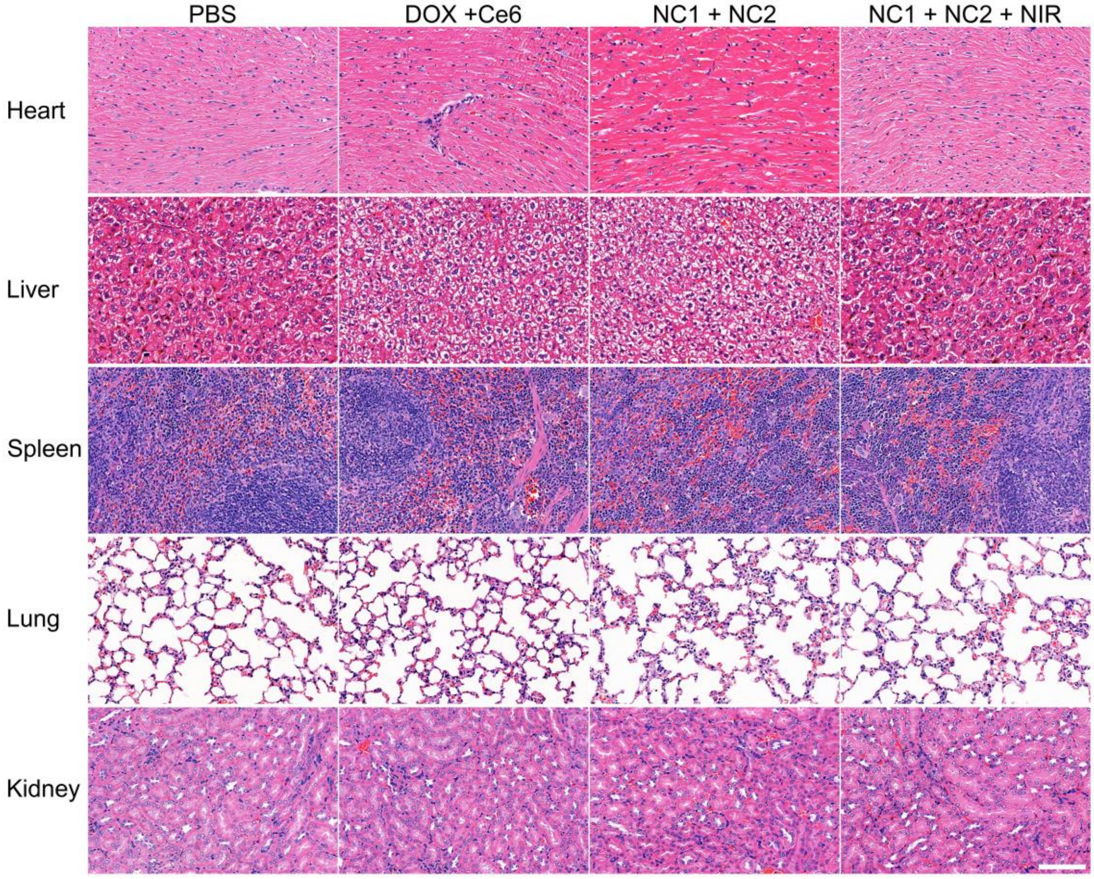

Figure S14. H&E analyses results of major organs dissected from A549 tumor-bearing nude mice after various treatments. Scale bar = 100 μm.

Table S1. DNA1 and DNA2 sequences with a different number of paired bases used to optimize NIR light-triggered DOX release.

Number of paired bases	DNA1	DNA2
10	TATAATGCCGC	GCGGCATTATT
20	TAATAATAATGCCGCCGCCG	CGGCGGCGGCATTATTATTA
30	AATAATAATAATAATGCCGCCGCCGCCGCC	GGCGGCGGCGGCGGCATTATTATTATTATT
40	ATAATAATAATAATAATAATGCCGCCGCCGCCGC CGCCGC	GCGGCGGCGGCGGCGGCGGCATTATTATTATTAT TATTAT
50	TAATAATAATAATAATAATAATAATGCCGCCGCC GCCGCCGCCGCCGCCG	CGGCGGCGGCGGCGGCGGCGGCGGCATTATTAT TATTATTATTATTATTA

Table S2. Pharmacokinetic parameters of NC1 and NC2.

	$T_{1/2}$ (h)	T_{max} (h)	C_{max} (ng/mL)	$AUC_{(0-\infty)}$ (h × ng/mL)	$MRT_{(0-\infty)}$ (h)	Cl_obs (mL/h)
**NC1**	2.97	0.33	14677.51	147954.59	18.49	1.35
**DOX**	0.62	0.33	5736.62	14252.56	7.46	14.03
**NC2**	4.79	0.33	4241.74	75928.53	27.55	2.63
**Ce6**	1.19	0.33	2177.60	8992.26	9.56	22.24
